# Retinofugal Projections from Melanopsin-Expressing Retinal Ganglion Cells Revealed by Intraocular Injections of Cre-Dependent Virus

**DOI:** 10.1371/journal.pone.0149501

**Published:** 2016-02-19

**Authors:** Anton Delwig, DeLaine D. Larsen, Douglas Yasumura, Cindy F. Yang, Nirao M. Shah, David R. Copenhagen

**Affiliations:** 1 Department of Ophthalmology, UCSF, San Francisco, California, United States of America; 2 Department of Anatomy, UCSF, San Francisco, California, United States of America; 3 Department of Physiology, UCSF, San Francisco, California, United States of America; NIH/NEI, UNITED STATES

## Abstract

To understand visual functions mediated by intrinsically photosensitive melanopsin-expressing retinal ganglion cells (mRGCs), it is important to elucidate axonal projections from these cells into the brain. Initial studies reported that melanopsin is expressed only in retinal ganglion cells within the eye. However, recent studies in *Opn4*-Cre mice revealed Cre-mediated marker expression in multiple brain areas. These discoveries complicate the use of melanopsin-driven genetic labeling techniques to identify retinofugal projections specifically from mRGCs. To restrict labeling to mRGCs, we developed a recombinant adeno-associated virus (AAV) carrying a Cre-dependent reporter (human placental alkaline phosphatase) that was injected into the vitreous of *Opn4*-Cre mouse eyes. The labeling observed in the brain of these mice was necessarily restricted specifically to retinofugal projections from mRGCs in the injected eye. We found that mRGCs innervate multiple nuclei in the basal forebrain, hypothalamus, amygdala, thalamus and midbrain. Midline structures tended to be bilaterally innervated, whereas the lateral structures received mostly contralateral innervation. As validation of our approach, we found projection patterns largely corresponded with previously published results; however, we have also identified a few novel targets. Our discovery of projections to the central amygdala suggests a possible direct neural pathway for aversive responses to light in neonates. In addition, projections to the accessory optic system suggest that mRGCs play a direct role in visual tracking, responses that were previously attributed to other classes of retinal ganglion cells. Moreover, projections to the zona incerta raise the possibility that mRGCs could regulate visceral and sensory functions. However, additional studies are needed to investigate the actual photosensitivity of mRGCs that project to the different brain areas. Also, there is a concern of "overlabeling" with very sensitive reporters that uncover low levels of expression. Light-evoked signaling from these cells must be shown to be of sufficient sensitivity to elicit physiologically relevant responses.

## Introduction

Melanopsin-expressing retinal ganglion cells (mRGCs) in the eye have been recently recognized as important mediators of non-image forming visual responses, such as circadian photoentrainment and pupillary light responses, in many mammalian species [1]. Published maps of central projections from mRGCs [2–5] have provided an important groundwork for formulating hypotheses related to the physiological and behavioral responses modulated by mRGCs. These previous studies relied on indirect labeling of mRGCs with an antibody against pituitary adenylate cyclase-activating peptide [2], or on low titer of intravitreal AAV-GFP [3], or on the direct labeling of all melanopsin-expressing cells using a reporter gene (*Opn4*-*lacZ*; [4]) or the genetic Cre-lox system (*Opn4*^*cre*^::*AP*^*loxP*^ and *Opn4*^*cre*^::*GFP*^*loxP*^; [5, 6]). The Cre-lox based genetic approach has been the most sensitive technique to reveal the diversity of mRGCs subtypes and their central targets. However, this approach also revealed extra retinal expression of floxed reporter genes in cells across many brain areas of *Opn4*^*cre*^ mice including cerebral cortex, thalamus and brainstem [5] that are not thought to express melanopsin. Melanopsin has also been recently found in the iris of mice using immunohistochemistry [7]. These findings complicate the use of melanopsin-driven genetic labeling techniques to identify retinofugal projections specifically from mRGCs

In this present study, we employed an alternative genetic method to specifically label mRGCs in the eye and to further increase the labeling of mRGCs with weak melanopsin expression. Delivery of a Cre-dependent reporter using a recombinant adeno-associated virus (AAV) has become a popular tool to label genetically defined cells. This method offers two advantages over the systemic Cre-lox genetic reporter approach. First, it does not reveal the historic pattern of expression thereby eliminating the report of transient expression in cells during early development. Second, it delivers multiple copies of the Cre-dependent reporter per infected cell thereby increasing the chance of recombination in cells with weak Cre expression. Therefore, we decided to further investigate the central targets of mRGCs in the brain by intravitreal injection of AAV carrying floxed human placental alkaline phosphatase (PLAP) reporter into *Opn4*^*cre*^ mice [5]. Here we present the results of these tracing studies.

## Methods

### Generating AAV-flex-plap

**Fig 1:**
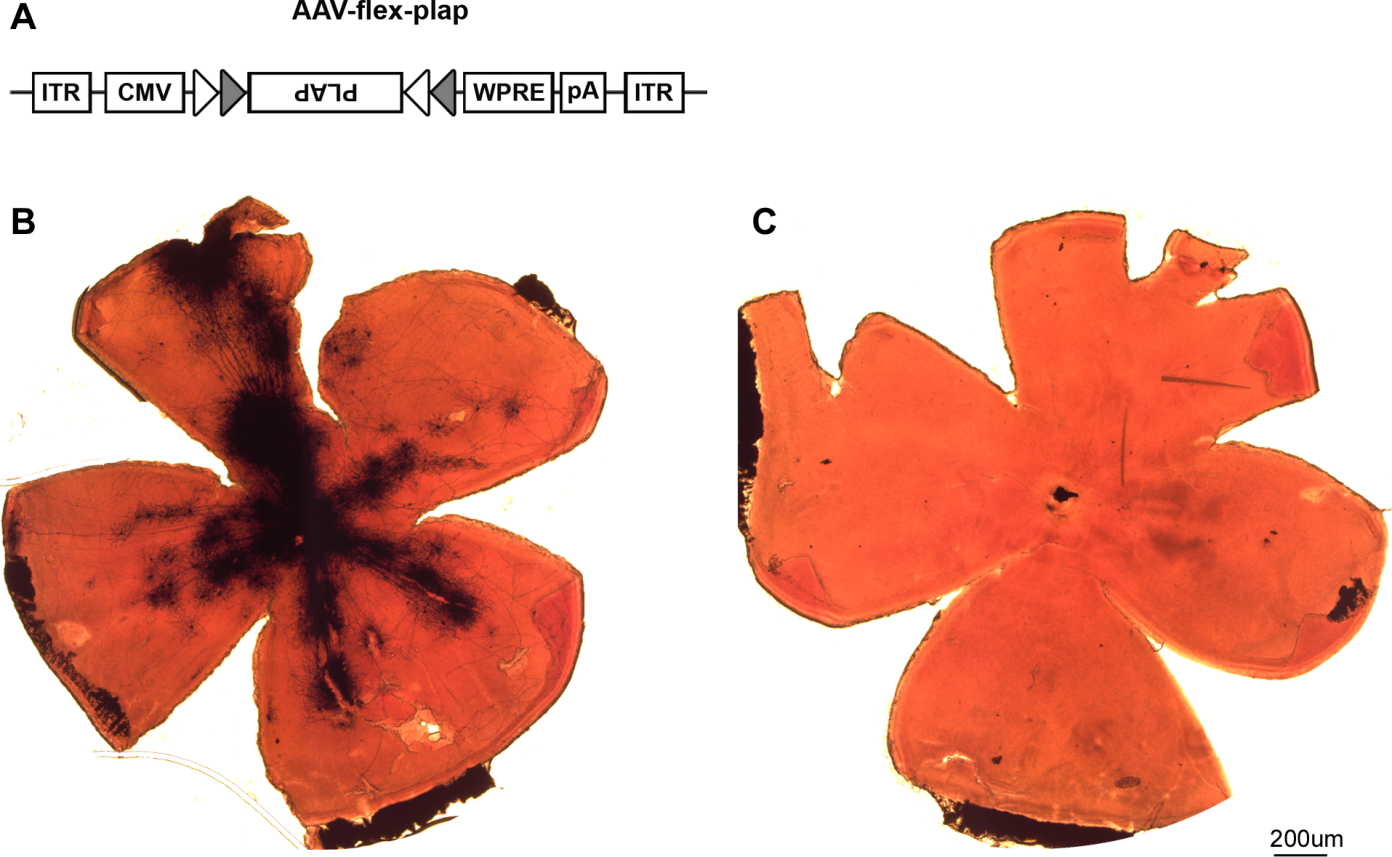
Selective labeling of mRGCs. (A) Map of the AAV-flex-plap vector that expresses alkaline phosphatase in a Cre-dependent manner (see methods for detailed description). (B, C) Representative flat-mount retinas from an *Opn4*^*cre*^ mouse that received intravitreal injection into the right eye. (B) Retina from the injected right eye. (C) Retina from the non-injected left eye. Scale bar: 200 um.

This virus was generated using standard sub-cloning with a modified pAAV-MCS backbone. The cDNA encoding human placental alkaline phosphatase (PLAP, NM_001632) was flanked by loxP (Fig 1A, open triangles) and lox2722 (Fig 1A, closed triangles) sites to yield the *flex-plap* transgene. This transgene was inserted in reverse orientation into a modified pAAV-MCS plasmid 3’ to a CMV promoter and 5’ to a woodchuck hepatitis virus post-transcriptional regulatory element (WPRE) and bovine growth hormone polyadenylation (pA) sequence to generate pAAV-flex-plap. High titer virus of serotype 2/1 (4x10^12^ IU/mL) was generated from this plasmid at the University of North Carolina, Chapel Hill Vector Core facility.

### Animals

Mice were housed in an AALAC-accredited pathogen-free animal facility with *ad libitum* access to food and water and with a 12-hour light-dark cycle with lights on at 7AM and off at 7PM. The University of California, San Francisco Institutional Animal Care and Use Committee (IACUC) specifically approved this study. The protocols, animal care procedures and the experimental methods meet all of the guidelines on the care and use of laboratory animals by the U.S. Public Health Service.

The following animals were used in this study: 1) C57BL/6J wild-type mice (Jackson Laboratory); 2) mice homozygous for *Opn4*^*cre*^ allele (gift from Samar Hattar [5]), which express Cre under the melanopsin (*Opn4*) promoter; and 3) mice homozygous for Ai14 allele (Jackson Lab [8]), which is a Cre-dependent tdTomato reporter. Mice were genotyped by PCR with allele-specific primers [5].

### Intravitreal injections

The age of the mice ranged from P38 to P96 at the time of injection. Mice were anesthetized with Isoflurane and topical administration of proparacaine (0.5%; Bausch & Lomb). The pupils were dilated by topical administration of phenylephrine (2.5%; Bausch & Lomb) and atropine sulfate (1%; Bausch & Lomb) eye drops. A 32-gauge Hamilton syringe was used to inject 2 microliters of AAV-flex-plap into the superior part of the vitreous of right eyes. A total of 13 injections were made (11 into *Opn4*^*cre*^ mice and 2 into C57BL/6J wild-type mice). No PLAP signal was detected in the retinas of wild-type mice. Visually detectible PLAP labeling was examined and quantified in the brains of 5 animals.

### Tissue processing for PLAP histochemistry

PLAP histochemistry was performed as previously described [9, 10]. Two to eight weeks after the intravitreal injection the mice were euthanized by CO_2_ and transcardially perfused with 10 ml HEPES-buffered saline (HBS; 8.2 g/l NaCl, 6 g/l HEPES, 0.1 g/l Na_2_HPO_4_, pH to 7.4 with NaOH) followed by 20 ml cold 4% paraformaldehyde (PFA) in HBS. All subsequent solutions were prepared with HBS unless otherwise noted. Brains and eyes were dissected and post-fixed in 4% PFA for 3–4 hours at 4°C. The brains were embedded in 3% agar and cut on a vibratome (Model 3000, Vibratome Company) into 100 μm sections. The retina was dissected from the fixed eyes and flat-mounted. Following rinsing in HBS, the endogenous alkaline phosphatase was heat-inactivated by incubation at 72°C for 1 hour. Tissue was then washed twice in buffer 1 (100 mM Tris pH 7.5, 150 mM NaCl) and then twice again in buffer 2 (100 mM Tris pH 9.5, 100 mM NaCl, 50 mM MgCl_2_). PLAP reporter was visualized by incubating tissue in buffer two with BCIP (5-bromo-4-chloro-3-indolyl phosphate, 0.2 mg/ml) and NBT (nitro blue tetrazolium, 1 mg/ml) at room temperature for 1 to 12 hours. PLAP reaction was monitored and stopped before background staining became excessive by rinsing 3 times in 1mM EDTA followed by post-fixation in 4% PFA for 1 hour. To remove background staining, the tissue was cleared by immersing in ethanol series (30%-70%-95%-100%-95%-70%-30%) for 1–3 minutes in each series and the subsequent wash in the HBS. Brain sections were counterstained (see below). Brain sections and whole mount retinas were mounted on microscope slides, briefly rinsed in distilled water and cover slipped using Aqua/Poly mount (Polysciences, Cat. # 18606).

### Counterstaining

To visualize brain nuclei, all brain sections were counterstained with ToPro3 (Life Technologies, Cat. # T3605), a fluorescent nuclear stain, at 1:5,000 dilutions. To better visualize thalamic nuclei, alternate sections were processed for Cytochrome Oxidase staining [11] by immersing slices in Cytochrome Oxidase staining solution (30 mg Cytochrome C, 20 mg Catalase, 50 mg DAB in 100 ml PBS) for 2–4 hours at room temperature.

## Results

### Labeling melanopsin retinal ganglion cells

We previously reported light-driven, melanopsin-dependent activation of neurons in the central amygdala and posterior thalamus in neonatal mice using immunolocalization of immediate early gene expression [12]. To further understand the contribution of melanopsin-expressing retinal ganglion cells (mRGCs) in the eye to non-image forming responses, we decided to look more carefully at projections of mRGCs to these and other brain areas. When we crossed *Opn4*^*cre*^ mice [5] with mice carrying floxed tdTomato reporter [8], we found tdTomato in neurons of the inner retina, presumed to be mRGCs. We also found tdTomato in many areas of the brain that are not known to be direct targets of retinal ganglion cells, such as the somatosensory cortex and cerebellum (S1 Fig). Similar extra retinal expression of *Opn4*^*cre*^ driven reporter was also observed by Ecker *et al*. [5].

To limit the expression of *Opn4*^*cre*^ driven reporter to retinofugal projections from mRGCs and to maximize the labeling of mRGCs with weak melanopsin expression, we created an adeno-associated virus (AAV) vector with floxed human placental alkaline phosphatase gene (AAV-flex-plap, Fig 1A; see methods). The intravitreal injection of AAV-flex-plap into *Opn4*^*cre*^ mice leads to robust labeling of many mRGCs around the site of injection (Fig 1B). The signal was always specific to the injected eye and was never observed in the contralateral, non-injected eye (Fig 1C). This result confirms that the virus did not spread outside the injected eye and that the labeling is specific to the virally encoded PLAP and not a result of endogenous alkaline phosphatase activity. We found that control injections of AAV-flex-plap into the vitreous of wild-type mice did not result in any labeling (n = 2, data not shown) thereby confirming that the PLAP signal is specific to *Opn4*^*cre*^ expressing retinal cells.

### Brain areas innervated by retinal mRGCs

**Fig 2:**
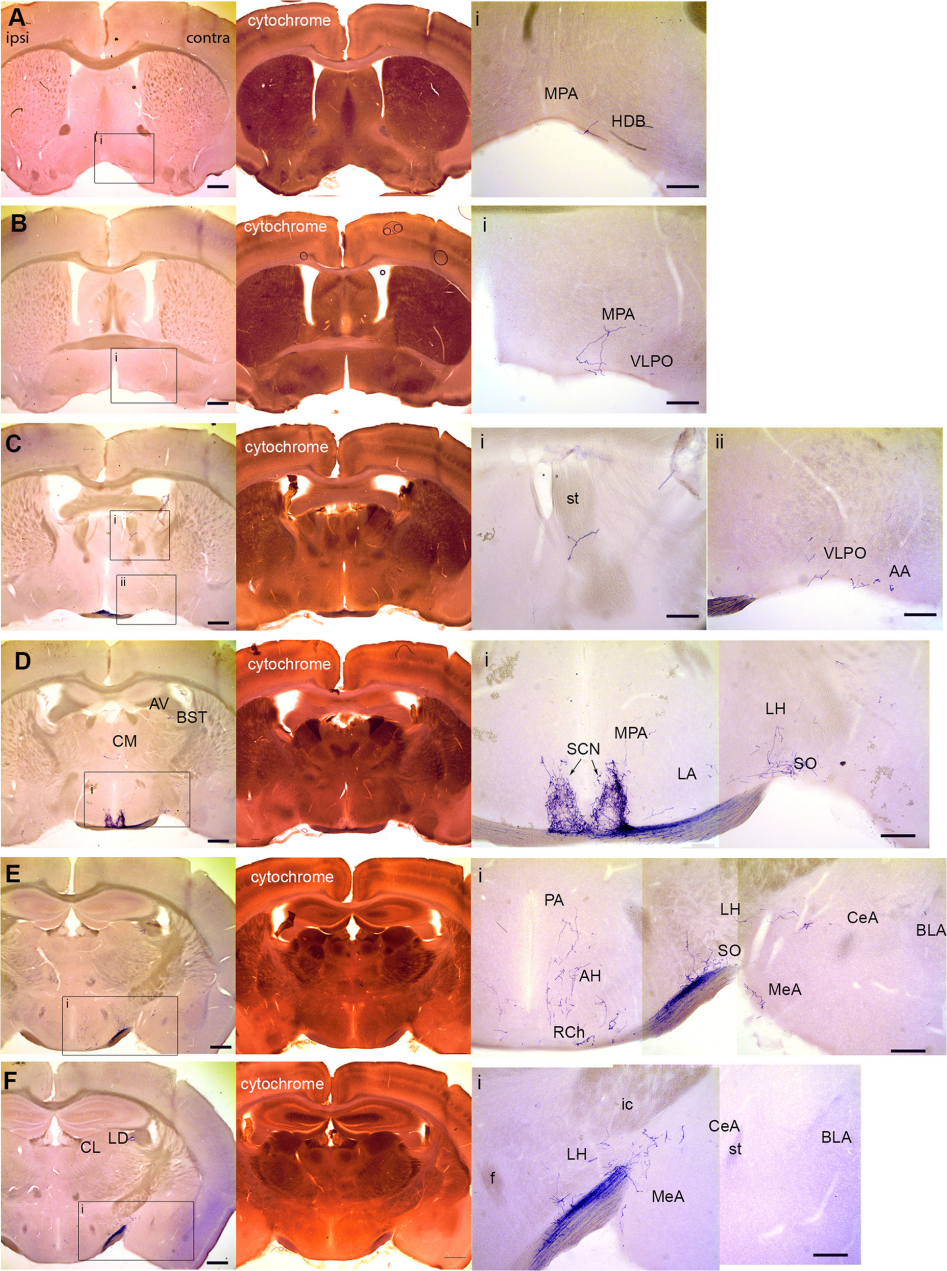
Central targets of mRGCs in the brain. Representative coronal sections (rostral to caudal) from the brains of *Opn4*^*cre*^ mice with intravitreal injection of AAV-flex-plap into the right eye. Left panels in each row are low-power images of brain sections processed to visualize alkaline phosphate. The next panel in each row is an image of an adjacent brain section stained for Cytochrome Oxidase. Some insets are composite montages of several images (D, E, F). See Table 1 for nomenclature. Scale bar: 200 **μ**m (inset, 100 **μ**m). Ipsilateral and contralateral sides of the brains are labeled. Note that HDB and MPA areas appear on the “contralateral”, right side of the section. Since these sections are rostral to the chiasm, one might think fibers in HDB and MPA should be on the ipsilateral side. However, we have traced these fibers back to the chiasm and find that they have indeed crossed over from the ipsilateral side and projected in the rostral direction.

**Fig 3:**
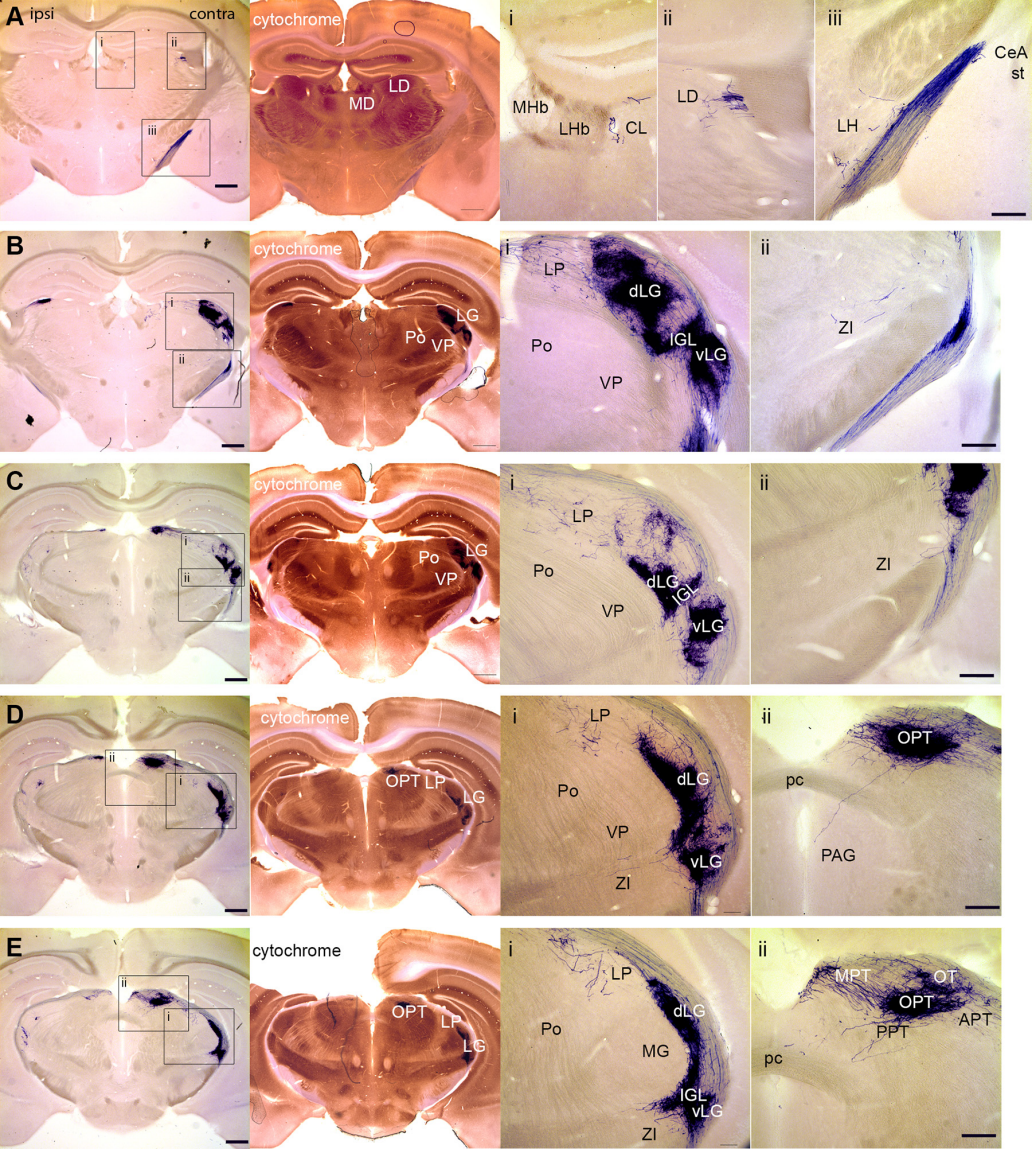
Central targets of mRGCs in the brain. Representative coronal sections (rostral to caudal) from the brains of *Opn4*^*cre*^ mice with intravitreal injection of AAV-flex-plap into the right eye. Left panels in each row are low-power images of brain sections processed to visualize alkaline phosphate. The next panel in each row is an image of an adjacent brain section stained for Cytochrome Oxidase. See Table 1 for nomenclature. Scale bar: 200 **μ**m (inset, 100 **μ**m).

Two to eight weeks after the intravitreal injection of AAV-flex-plap, the brains were sectioned coronally and processed to visualize PLAP. A representative coronal series is presented in Figs 2 and 3. The following sections describe our findings (summarized in Tables 1 and 2). For each case analyzed the entire series of sections was scored for the presence or absence of PLAP-positive axons in each brain area. Adjacent sections processed for cytochrome oxidase staining and ToPro3 counterstains were used to identify architectonic boundaries in the sections. The Mouse Brain in Stereotaxic Coordinates [13] and Allen Mouse Brain Atlas [14] were used to identify regions and nuclei in the stained sections. For AOS anatomy we also referred to Giolli et al. [15].

**Table 1:** Abbreviations used in the text and in the figures.

Anatomical Area	Abbreviation
anterior amygdala	AA
anterior hypothalamic area	AH
anterior pretectal nuclei	APT
anteroventral nucleus	AV
bed nucleus of the stria terminalis	BST
central amygdala	CeA
central lateral nucleus	CL
central medial nucleus	CM
dorsal terminal nucleus of the accessory optic tract	DT
dorsal lateral geniculate nucleus	dLG
internal capsule	ic
intergeniculate leaflet	IGL
lateral dorsal nucleus	LD
lateral hypothalamus	LHA
lateral habenular nucleus	LHb
Lateral posterior nucleus	LP
lateral preoptic area	LPO
lateral terminal nucleus of the accessory optic tract	LT
medial dorsal nucleus	MD
medial amygdala	MeA
medial geniculate nucleus	MG
medial habenular nucleus	MHb
medial preoptic area	MPO
medial pretectal nuclei	MPT
medial terminal nucleus of the accessory optic tract	MT
horizontal limb of the diagonal band	HDB
nucleus of the optic tract	NOT
Olivary pretectal nucleus	OPT
periaqueductal gray	PAG
posterior commissure	Pc
Posterior thalamic nucleus	Po
posterior pretectal nuclei	PPT
paraventricular hypothalamus	PA
retrochiasmatic nucleus	RCh
Superior colliculus	SC
suprachiasmatic nucleus	SCN
supraoptic nucleus	SO
stria terminalis	st
ventral lateral geniculate nuclei	vLG
ventrolateral preoptic area	VLPO
ventral posterior thalamic nucleus	VP
zona incerta	ZI

**Table 2:** Compilation of frequency of innervation by PLAP-positive fibers.

	# Of cases	% Of cases
**Basal Forebrain and Hypothalamus**		
NDB	1	20%
MPO	3	60%
LPO	3	60%
VLPO	2	40%
SO	5	100%
BST	4	80%
LHA	5	100%
RCh	5	100%
SCN	5	100%
AA	5	100%
MeA	5	100%
CeA	5	100%
**Thalamus and Habenula**		
CM	2	40%
LD	5	100%
CL	5	100%
MD	1	20%
LP	5	100%
dLG	5	100%
vLG	5	100%
IGL	5	100%
ZI	5	100%
**Midbrain**		
PAG	3	60%
OP	5	100%
APN	5	100%
MPT	5	100%
PPT	5	100%
NOT	5	100%
SC	5	100%
DT	5	100%
LT	5	100%
MT	5	100%

#### Basal forebrain and hypothalamus

The most rostral targets of mRGCs that we observed in the mouse brain were the horizontal limb of the diagonal band (HDB, 1/5), the lateral preoptic area (LPO, 3/5) and the medial preoptic area (MPO, 3/5; Fig 2A and 2B). The sparse labeling was always on the contralateral side. Further caudally, single mRGCs axons were found in the ventrolateral preoptic area (VLPO, 2/5) and the anterior amygdala (AA, 5/5), again always on the contralateral side (Fig 2B and 2C). Dense bilateral staining was observed in the suprachiasmatic nucleus (SCN, 5/5; Fig 2D). Caudal to the SCN, midline areas tended to be bilaterally innervated whereas lateral targets were innervated contralaterally. Sparse bilateral labeling was seen in retrochiasmatic nucleus (RCh, 5/5; Fig 2E). Sparse contralateral projections were observed in the medial preoptic area (MPO, 3/5), lateral hypothalamus (LHA, 5/5), supraoptic nucleus (SO, 5/5) and medial amygdala (MeA, 5/5; Fig 2D–2F). A novel finding of this study was the observation of sparse innervation of the central amygdala (CeA, 5/5; Fig 2E and 2F).

#### Thalamus and habenula

Single mRGC axons were observed innervating contralateral bed nucleus of the stria terminalis (BST, 4/5) and bilateral central medial nucleus (CM, 2/5; Fig 2C and 2D). More caudally, mRGCs axons were found in the contralateral lateral dorsal nucleus (LD, 5/5) and central lateral nucleus (CL, 5/5; Fig 3A). We did not observe projections into the lateral habenula (LHb). The terminals of mRGCs were localized in the nearby CL (Fig 3A), with a single case that also extended into the medial dorsal nucleus (MD, 1/5). Lateral posterior nucleus (LP, 5/5) received contralateral projections from mRGCs of medium density (Fig 3B–3E). We observed dense bilateral innervation of the dorsal lateral geniculate nucleus (dLG, 5/5) and dense mostly contralateral innervation of the ventral lateral geniculate nuclei (vLG, 5/5) and intergeniculate leaflet (IGL, 5/5; Fig 3B–3E). Sparse contralateral innervation was observed in the zona incerta (ZI, 5/5; Fig 3B–3E). We did not observe projections from mRGCs to the ventral posterior nucleus (VP) or posterior thalamic nuclear group (Po; Fig 3B–3E).

#### Midbrain

Sparse contralateral innervation was observed in the periaqueductal gray (PAG, 3/5; Fig 3D). Olivary pretectal nucleus (OPT, 5/5) received dense innervation by mRGCs with most projections going to the contralateral side (Fig 3C–3E). Other visual pretectal nuclei received bilateral innervation of medium density. They included the anterior, medial and posterior pretectal nuclei (APT, MPT, and PPT, 5/5) and the nucleus of the optic tract (NOT, 5/5; Fig 3D and 3E).

#### Accessory optic system and the superior colliculus

**Fig 4:**
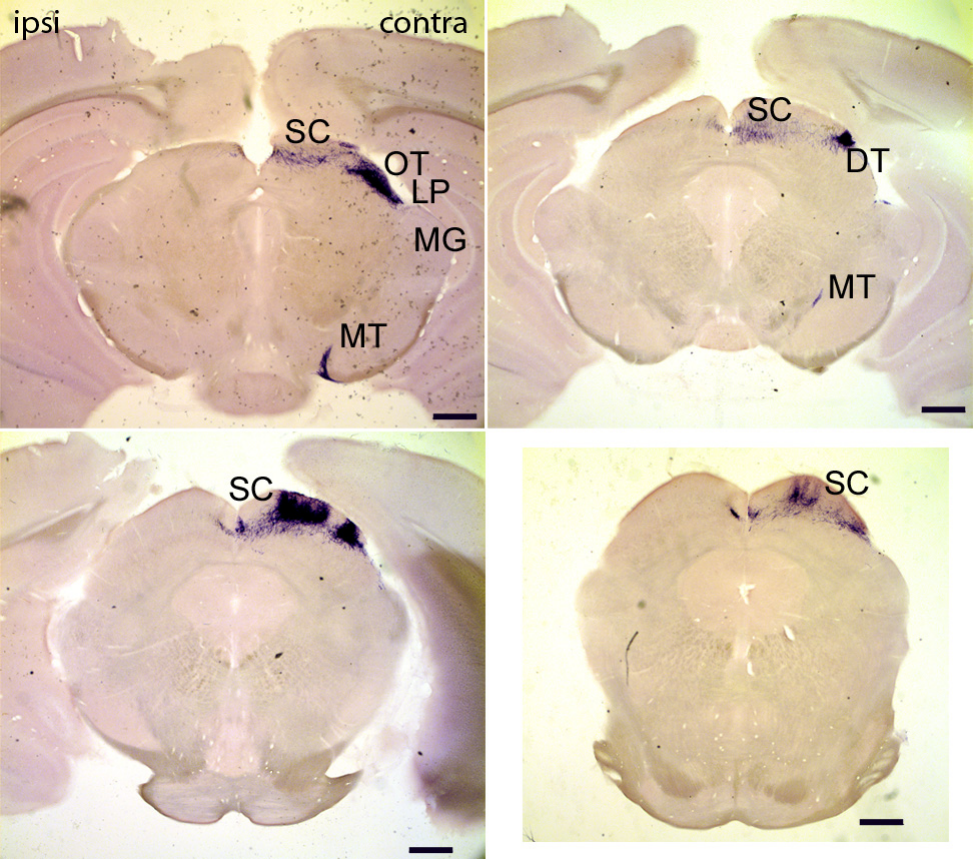
Projections of mRGCs to accessory optic system. Four representative coronal sections show the projections of mRGCs to the accessory optic system and the superior colliculus. Note these sections contained dark crystal-like puncta we believe are salt crystals that were not adequately washed out during slide preparation. Based on color and positional differences, we felt comfortable differentiating between these artifacts and the purple/blue PLAP reaction products. Scale bar: 200 **μ**m.

Another novel finding was the observation of dense contralateral innervation by mRGCs of the accessory optic nuclei including dorsal, lateral, and medial terminal nuclei (DT, LT, and MT 5/5; Fig 4). The superior colliculus (SC, 5/5) received dense, mostly contralateral, innervation (Fig 4).

## Discussion

In the present study, we used viral delivery of a Cre-dependent reporter to label melanopsin-expressing retinal ganglion cells (mRGCs) in the mouse eye and to determine what central targets in the brain they innervate. We found that mRGCs innervate multiple nuclei in the basal forebrain, hypothalamus, amygdala, thalamus and midbrain. Midline structures tended to be bilaterally innervated whereas the lateral structures received mostly contralateral innervation. These results are in accord with the previously published results [2–5]. The following sections highlight the novel targets and discrepancies with the previous studies. A caveat of the intravitreal viral delivery of the reporter is that this technique does not label all mRGCs in the eye. It labels only a subset of mRGCs near the site of injection (Fig 1B). Therefore, this technique only samples the projections by mRGCs from one region of the retina; there might be additional brain regions innervated by mRGCs that were not labeled by our injections. Another caveat is that PLAP labeling in various brain areas does not necessarily represent synapses in these regions; PLAP labeled fibers could represent fibers of passage.

A critical question is whether we were viewing axon terminals targeted to different brain regions or might the PLAP-positive fibers be axons of passage? Based on our impression that the axons ended in a particular region we assumed this was an area targeted by mRGCs. However to substantiate this assertion we would have to do high-resolution microscopy and antibody labeling. Axon terminals should be enriched for synaptic proteins such as VGLUT2 or synapsin, or show structural specializations usually associated with synaptic boutons.

### Central amygdala

A novel finding of this study is sparse but consistent innervation of the central amygdala (Fig 2E and 2F). These findings suggest that the light-induced neural activation in CeA of the neonatal mice [12] may be due to direct activation of neurons in CeA by mRGCs. The extent of direct vs. indirect effect of mRGCs on neural activation in CeA remains unknown. We speculate that if this retina-amygdala pathway is conserved in humans, it may be the neural mechanism of aversive responses to light in neonatal and preterm infants [16, 17].

### Zona incerta

We found consistent projections of mRGCs to the zona incerta (Fig 3B–3E). It is one of the limbic nodes and is implicated in the regulation of water and food intake, reproductive behaviors and cardiovascular activity [18]. Additionally, the zona incerta is involved in processing pain by controlling the transmission of signals from the spinothalamic tract to the posterior thalamus [19]. In primates, zona incerta is also implicated in controlling saccades via GABAergic projections to the superior colliculus [20]. Altogether, our findings suggest that mRGCs may play role in regulating these non-image forming responses.

### Posterior thalamus

An electrophysiological study by Noseda *et al*. [21] of light-responsive neurons in LD, LP, VP and Po in rats suggested a direct input from mRGCs to these thalamic regions. We were able to detect projections from mRGCs to LD and LP but not to VP or Po (Fig 3A–3E) in mice. The observed lack of innervation in VP and Po does not necessarily prevent neurons in these brain areas from receiving light signals originating from mRGCs. The dendritic fields of VP and Po neurons are large enough (350 **μ**m; [22]) to receive direct visual input from nearby visual thalamic areas that are heavily innervated by mRGCs. Additionally, the absence of direct innervation of Po and VP that we report here could reflect differences between mice and rats.

### Accessory optic system (AOS)

An unexpected finding of our study is the heavy innervation of the accessory optic system by mRGCs. We saw dense projections to all AOS nuclei including OT, DT and MT (Figs 3E and 4) suggesting that mRGCs contribute to tracking responses. Consistent with this suggestion, an M4 subtype of mRGCs was recently found to contribute to the contrast sensitivity of the optokinetic response [23]. However, in contrast to our findings, Hoxd10 retinal ganglion cells, which include many types of AOS projecting directionally selective cells in the retina [24], do not express melanopsin and are not intrinsically photosensitive as determined by immunolabeling with anti-melanopsin antibody and whole-cell electrophysiology (personal communication with Maureen Stabio and David Berson). Additionally, the previous study by Ecker *et al*. [5] using the same *Opn4*^*cre*^ mouse strain and a genetic reporter did not detect any signal in the AOS. One possible resolution of the apparent disparity between our results and that of Ecker et al. is that AAV-mediated delivery of Cre-dependent reporter may result in multiple copies of viral genomes per infected cell, thereby increasing the chance of detecting cells with low level of melanopsin expression. It is possible that this increased sensitivity revealed novel AOS projecting subtypes of mRGCs that were not previously described. There are a number of additional experiments that need be carried out to further investigate the contribution of mRGCs to visual tracking: (a) analysis of labeled mRGCs to see if they include directionally selective retinal ganglion cells; and (b) retrograde labeling of retinal ganglion cells projecting to MT and OT nuclei followed by analysis of melanopsin expression and of their intrinsic photosensitivity.

### Retinal photoreceptors

We found examples of PLAP positive cones in pAAV-flex-plap transfected retinas (S2 Fig). These results corroborate previous studies reporting melanopsin-driven reporter expression in photoreceptors (Ecker et al. 2010). A critical question from a developmental standpoint is when is *Opn4* active? In this present study we find PLAP expression in retinas injected after postnatal day 30. Whether melanopsin photopigment contributes to detection of light in the Opn4-expressing photoreceptors will require further study.

Moreover, our ability to observe PLAP-positive photoreceptors is informative with respect to finding an AAV serotype (2/1) that is capable of infecting photoreceptors when injected into the vitreous. This has importance for devising gene therapy interventions in outer retinal degenerative diseases. Viral transfection of rods and cones following an injection different AAV serotypes into the vitreous is uncommon. Of 7 different serotypes tested by Hellstrom *et al*. [25] only AAV2/3 and AAV2/5 seemed efficacious for transducing rods and cones. Our findings show that AAV2/1 can also transduce photoreceptors, suggesting AAV2/1 has properties similar to AAV2/3 or AAV2/5. Further study is required to quantitatively compare transduction percentages of these different serotypes.

Two issues using AAV based markers deserve consideration. 1. An important concern that arises from the use of highly sensitive reporters is the relationship between the labeling and the physiologically relevant level of melanopsin expression. As labeling techniques become progressively more sensitive, with an ability to mark ever-lower levels of expression, a question arises as at what point the labeling becomes excessive and is not physiologically relevant. It is possible that our technique labeled RGCs with low levels of melanopsin expression that may not be sufficient to confer intrinsic photosensitivity or a significant biological function. Further studies are needed to show that the labeled mRGCs are indeed intrinsically photosensitive.

2. An important question that arises is whether the virus we used transfected selective classes of mRGC. The intersectional genetic strategy used by Ecker *et al*. [5] showed that *Opn4*-cre was virtually ubiquitously expressed, however, we cannot assess whether our PLAP-flex-plap virus infected all subtypes of mRGC. Differences in the density of staining in target regions identified by intravitreal injections of a rAAV-GFP (Gooley, *et al*, [3]) and ours is consistent with the notion that different viruses may not infect all classes of ipRGC. Hellstrom e*t al*. [25] illustrated evidence for selective transduction of subsets of single classes of retinal cell by different AAV serotypes. In conclusion we cannot say whether selective classes of ipRGC were transfected by our AAV-flex-plap injections.

In summary, our findings highlight sparse but diffuse innervation of multiple limbic areas in the brain suggesting that light activation of mRGCs may contribute to regulation of multiple homeostatic responses in the animal including sleep, feeding, reproductive behaviors, cardiovascular function, alertness, mood, pain and memory. Additionally, projections to the accessory optic system suggest that mRGCs play role in saccades and visual tracking, responses that were previously attributed to other classes of retinal ganglion cells.

## Supporting Information

S1 Fig Extra retinal expression of *Opn4*^*cre*^ driven reporter. Representative examples of *Opn4*^*cre*^ -driven expression of Ai14, a floxed tdTomato fluorescent reporter, in the brain of P21 mouse (*Opn4*^*cre*^::Ai14). Widespread expression of tdTomato is observed in numerous areas including (A) the somatosensory cortex, (B) thalamus, and (C) cerebellum. Scale bar: 100 um.
(TIFF)

S2 Fig AAV-mediated labeling of *Opn4*^*cre*^ cells in the retina. Three representative retinal slices from *Opn4*^*cre*^ mouse with intravitreal injection of AAV-flex-plap. PLAP staining was maximized to reveal finer details of PLAP labeling in a sparse population of cones, albeit at the expense of saturating PLAP signal in the retinal ganglion cells and in the inner plexiform layer. Abbreviations: GC—ganglion cell layer; IPL—inner plexiform layer; INL—inner nuclear layer; OPL—outer plexiform layer; ONL—outer nuclear layer; OS—outer segments.
(TIFF)

## References

[1] Do MTH, Yau K-W. Intrinsically photosensitive retinal ganglion cells. Physiol Rev. 2010; 90: 1547–1581. (2010) 10.1152/physrev.00013.2010 20959623 PMC4374737

[2] Hannibal J, Fahrenkrug J. Target areas innervated by PACAP-immunoreactive retinal ganglion cells. Cell Tissue Res. 2004; 316: 99–113. 10.1007/s00441-004-0858-x 14991397

[3] Gooley JJ, Lu J, Fischer D, Saper CB. A broad role for melanopsin in nonvisual photoreception. J Neurosci 2003; 23: 7093–7106. 12904470 10.1523/JNEUROSCI.23-18-07093.2003 PMC6740653

[4] Hattar S, Kumar M, Park A, Tong P, Tung J, Yau KW, et al Central projections of melanopsin-expressing retinal ganglion cells in the mouse. J Comp Neurol. 2006; 497: 326–349. 10.1002/cne.20970 16736474 PMC2885916

[5] Ecker JL, Dumitrescu ON, Wong KY, Alam NM, Chen S-K, LeGates T, et al Melanopsin-expressing retinal ganglion-cell photoreceptors: cellular diversity and role in pattern vision. Neuron 2010; 67: 49–60. 10.1016/j.neuron.2010.05.023 20624591 PMC2904318

[6] Brown TM, Gias C, Hatori M, Keding SR, Semo M, Coffey PJ, et al Melanopsin contributions to irradiance coding in the thalamo-cortical visual system. PLoS Biol. 2010 8(12). 10.1371/journal.pbio.1000558 PMC2998442 21151887

[7] Xue T, Do MTH, Riccio A, Jiang Z, Hsieh J, Wang HC, et al Melanopsin signalling in mammalian iris and retina. Nature 2011; 479: 67–73. 10.1038/nature10567 22051675 PMC3270891

[8] Madisen L, Zwingman TA, Sunkin SM, Oh SW, Zariwala HA, Gu H, et al A robust and high-throughput Cre reporting and characterization system for the whole mouse brain. Nat Neurosci. 2010; 13: 133–140. 10.1038/nn.2467 20023653 PMC2840225

[9] Shah NM, Pisapia DJ, Maniatis S, Mendelsohn MM, Nemes A, Axel R. Visualizing sexual dimorphism in the brain. Neuron 2004; 43: 313–319. 10.1016/j.neuron.2004.07.008 15294140

[10] Yang CF, Chiang MC, Gray DC, Prabhakaran M, Alvarado M, Juntti SA, et al Sexually dimorphic neurons in the ventromedial hypothalamus govern mating in both sexes and aggression in males. Cell 2013; 153: 896–909. 10.1016/j.cell.2013.04.017 23663785 PMC3767768

[11] Wong-Riley MT, Carroll EW. Quantitative light and electron microscopic analysis of cytochrome oxidase-rich zones in V II prestriate cortex of the squirrel monkey. J Comp Neurol. 1984; 222: 18–37. 10.1002/cne.902220103 6321563

[12] Delwig A, Logan AM, Copenhagen DR, Ahn AH. Light Evokes Melanopsin-Dependent Vocalization and Neural Activation Associated with Aversive Experience in Neonatal Mice. PloS One 2012; 7: e43787 10.1371/journal.pone.0043787 23028470 PMC3441538

[13] Franklin KBJ, Paxinos G. Paxinos and Franklin’s the mouse brain in stereotaxic coordinates Fourth edition Amsterdam: Academic Press, an imprint of Elsevier; 2013.

[14] Allen Mouse Brain Atlas Website: © 2015 Allen Institute for Brain Science. Available: http://mouse.brain-map.org.

[15] Giolli RA, Blanks RH, Lui F. The accessory optic system: basic organization with an update on connectivity, neurochemistry, and function. Prog Brain Res. 2006;151:407–40. 16221596 10.1016/S0079-6123(05)51013-6

[16] Shogan MG, Schumann LL. The effect of environmental lighting on the oxygen saturation of preterm infants in the NICU. Neonatal Netw. NN 1993;12: 7–13. 8350854

[17] Lasky RE, Williams AL. Noise and light exposures for extremely low birth weight newborns during their stay in the neonatal intensive care unit. Pediatrics 2009; 123: 540–546. 10.1542/peds.2007-3418 19171620

[18] Mitrofanis J. Some certainty for the “zone of uncertainty”? Exploring the function of the zona incerta. Neuroscience 2005;130: 1–15. 10.1016/j.neuroscience.2004.08.017 15561420

[19] Masri R, Quiton RL, Lucas JM, Murray PD, Thompson SM, Keller A, et al Zona incerta: a role in central pain. J Neurophysiol. 2009;102: 181–191. 10.1152/jn.00152.2009 19403748 PMC2712264

[20] Ma TP. Saccade-related omnivectoral pause neurons in the primate zona incerta. Neuroreport 1996; 7: 2713–2716. 8981453 10.1097/00001756-199611040-00061

[21] Noseda R, Kainz V, Jakubowski M, Gooley JJ, Saper CB, Digre K, et al A neural mechanism for exacerbation of headache by light. Nat Neurosci. 2010;13(2):239–45. 10.1038/nn.2475 20062053 PMC2818758

[22] Noseda R, Jakubowski M, Kainz V, Borsook D, Burstein R. Cortical projections of functionally identified thalamic trigeminovascular neurons: implications for migraine headache and its associated symptoms. J Neurosci. 2011; 13:239–45. doi: 10.1038/nn.2475 10.1523/JNEUROSCI.3285-11.2011 PMC3501387 21976505

[23] Schmidt TM, Alam NM, Chen S, Kofuji P, Li W, Prusky GT, et al A role for melanopsin in alpha retinal ganglion cells and contrast detection. Neuron 2014; 82: 781–788. 10.1016/j.neuron.2014.03.022 24853938 PMC4083763

[24] Dhande OS, Estevez ME, Quattrochi LE, El-Danaf RN, Nguyen PL, Berson DM, et al Genetic dissection of retinal inputs to brainstem nuclei controlling image stabilization. J Neurosci 2013; 33: 17797–17813. 10.1523/JNEUROSCI.2778-13.2013 24198370 PMC3818553

[25] Hellström M, Ruitenberg MJ, Pollett MA, Ehlert EM, Twisk J, Verhaagen J, et al Cellular tropism and transduction properties of seven adeno-associated viral vector serotypes in adult retina after intravitreal injection. Gene Ther. 2009 16:521–32. 10.1038/gt.2008.178 19092858

