# Supplementary figures and images for: Retinofugal Projections from Melanopsin-Expressing Retinal Ganglion Cells Revealed by Intraocular Injections of Cre-Dependent Virus
Source: PLoS One. 2016 Feb 19;11(2):e0149501. doi: 10.1371/journal.pone.0149501 (PMC4764510; doi:10.1371/journal.pone.0149501)

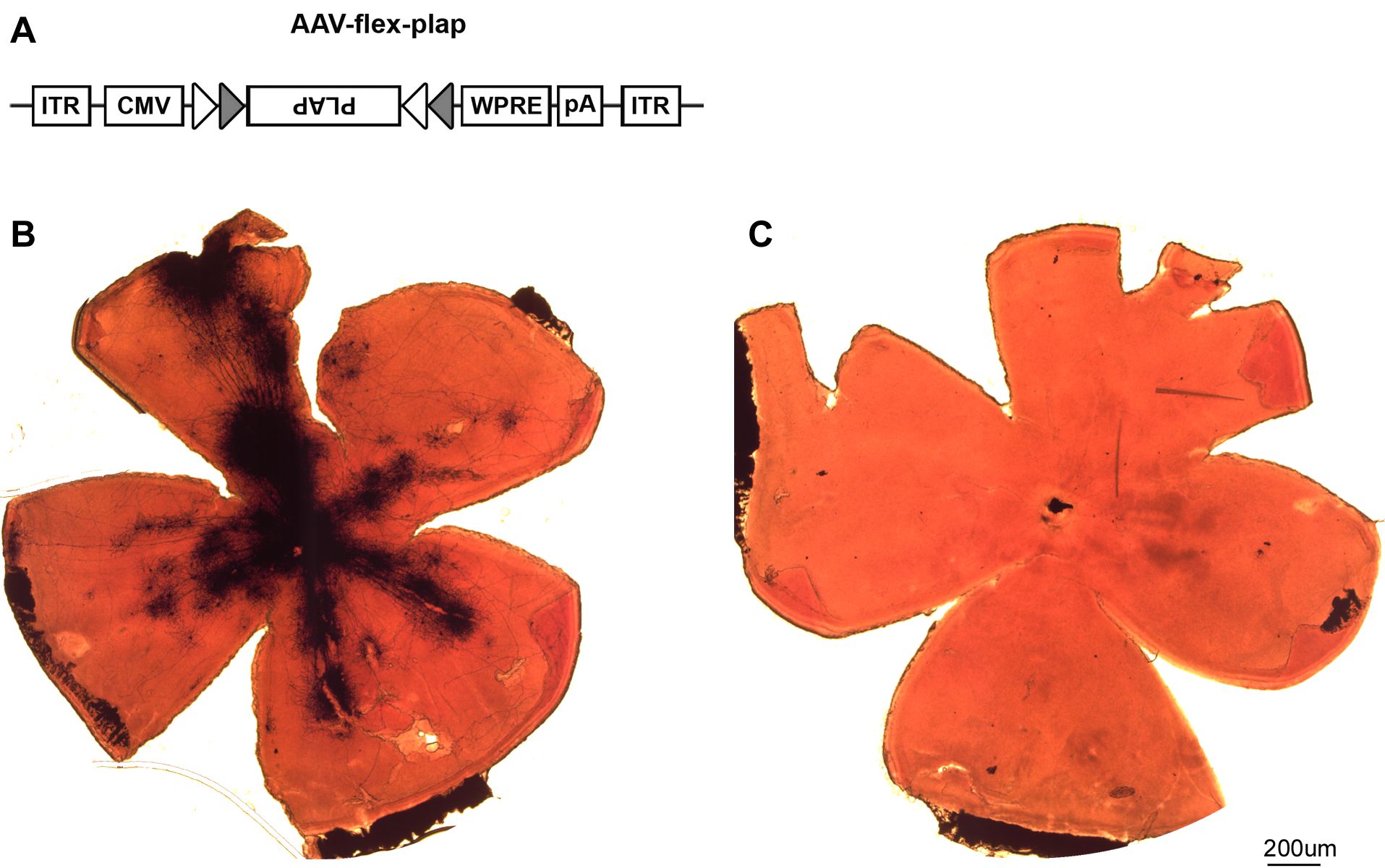

Supplement: S1 Fig — Representative examples of Opn4cre -driven expression of Ai14, a floxed tdTomato fluorescent reporter, in the brain of P21 mouse (Opn4cre::Ai14). Widespread expression of tdTomato is observed in numerous areas including (A) the somatosensory cortex, (B) thalamus, and (C) cerebellum. Scale bar: 100 um. (TIFF) [file pone.0149501.s001.tiff]

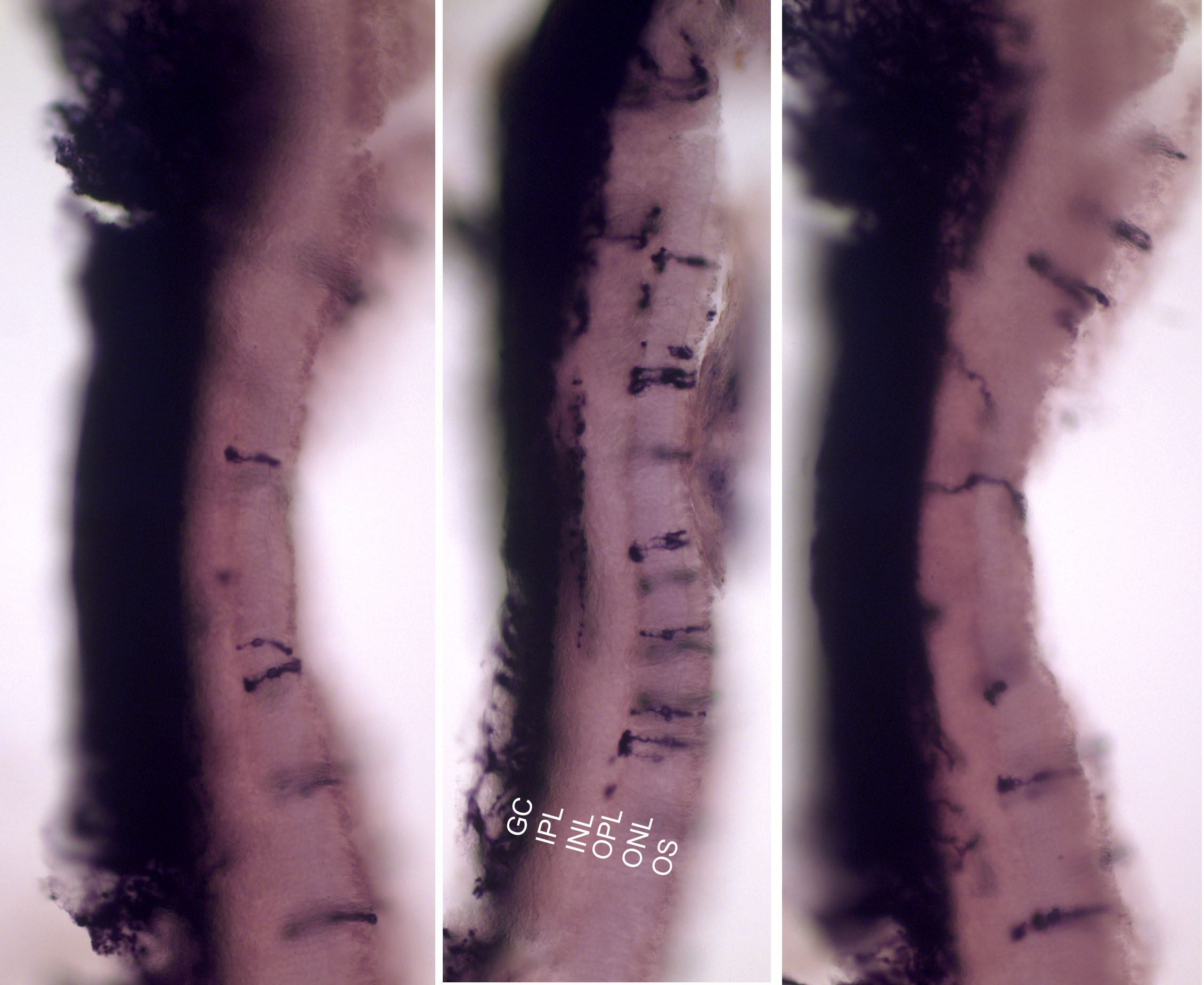

Supplement: S2 Fig — Three representative retinal slices from Opn4cre mouse with intravitreal injection of AAV-flex-plap. PLAP staining was maximized to reveal finer details of PLAP labeling in a sparse population of cones, albeit at the expense of saturating PLAP signal in the retinal ganglion cells and in the inner plexiform layer. Abbreviations: GC—ganglion cell layer; IPL—inner plexiform layer; INL—inner nuclear layer; OPL—outer plexiform layer; ONL—outer nuclear layer; OS—outer segments. (TIFF) [file pone.0149501.s002.tiff]
